# How Treatment Motivation Predicts Favorable Outcomes in Interdisciplinary Multimodal Pain Treatment Among Patients with Chronic Primary Pain

**DOI:** 10.1007/s10880-023-09958-0

**Published:** 2023-04-20

**Authors:** Alina Scheidegger, Juan Martín Gómez Penedo, Larissa Tatjana Blättler, Selma Aybek, Nina Bischoff, Martin grosse Holtforth

**Affiliations:** 1 Psychosomatic Medicine, Department of Neurology, Inselspital, Bern University Hospital, University of Bern, Bern, Switzerland; 2 Facultad de Psicología, CONICET Universidad de Buenos Aires, Buenos Aires, Argentina; 3 Department of Psychology, University of Bern, Bern, Switzerland

**Keywords:** Treatment motivation, Hopelessness, Chronic primary pain, Interdisciplinary multimodal pain treatment

## Abstract

As motivation for psychological treatment at intake has been shown to predict favorable outcomes after an inpatient stay, this study aimed to further characterize the different components of psychological treatment motivation that predict favorable treatment outcomes. 294 inpatients with chronic primary pain participating in an interdisciplinary multimodal pain treatment in a tertiary psychosomatic university clinic completed a battery of psychological questionnaires at intake and discharge. Treatment motivation was assessed at intake using the scales of the FPTM-23 questionnaire, while pain intensity, pain interference, anxiety, and depression were assessed both at intake and discharge*.* After treatment, pain intensity, pain interference, anxiety, and depression were significantly reduced. While higher levels on the FPTM-23 scale of *suffering* predicted smaller decreases in anxiety after treatment, higher scores on the scale of *hope*, i.e., lower levels of hopelessness, predicted lower levels of pain interference, anxiety, and depression after treatment. None of the scales of treatment motivation predicted pain intensity levels after treatment. Above and beyond providing symptom relief, reducing hopelessness and fostering hope regarding the treatment process and outcome might help clinicians treat patients with chronic primary pain more effectively.

## Introduction

Chronic pain is a global health problem that causes considerable suffering and affects almost every aspect of a person’s life (Gardner & Sachdeva, 2019; Mills et al., 2019; Velly & Mohit, 2018). Due to its complex nature, the biopsychosocial approach of interdisciplinary multimodal pain treatment is particularly suitable for treating chronic pain (Kaiser et al., 2018). Interdisciplinary multimodal pain treatment combines different therapy methods like psychological treatment, physiotherapy, relaxation techniques, occupational therapy, medical training therapy, workplace training/training for everyday living, as well as interventional pain therapy (Kaiser et al., 2018). While the treatment also aims to reduce pain intensity and enhance patients’ emotional well-being, the interventions primarily aim to increase patients’ physical and psychological functioning despite their chronic pain (Kaiser et al., 2018).

Oftentimes, psychoeducation is needed to shift patients’ focus from desirable changes in somatic aspects like pain intensity to more realistic and favorable changes in pain interference and psychological well-being. In psychoeducation, as an early part of psychological treatment, therapists aim to convey the biopsychosocial model of pain as the basis for understanding and treating chronic pain (Arnold et al., 2014). Psychoeducation provides an essential context for identifying potential biological, psychological, and social factors maintaining and aggravating chronic pain together with the patient (Arnold et al., 2014; Kaiser et al., 2018). A better understanding of maintaining factors for chronic pain allows clinicians and patients to work on changes in thoughts and behaviors which might, in turn, alleviate pain interference and psychological distress. Moreover, a better understanding of influencing factors might motivate patients to engage in other treatment modalities, i.e., physiotherapy and occupational therapy.

Although professionals consider psychological treatment crucial in chronic pain treatment, patients may be more or less motivated to actively engage in psychological treatment (Kaiser et al., 2018; Westermann et al., 2019). The global construct of therapy motivation subsumes several subjective factors directly affecting patients’ motivation to engage in treatment (Drieschner et al., 2004).

Generally, *suffering* is often viewed as the driving force of treatment since it fuels the desire to change something and can be seen as a precondition for problem awareness (Schulte, 2015). Suffering is composed of various negative experiences associated with the illness such as symptom distress, feelings of deviation from the norm, and related impairments (Schulte, 2015). Thus, patients’ primary motive at the beginning of therapy is to reduce the negative aspects or consequences of their suffering (Schulte, 2015). Moreover, the *hope* of reducing their symptoms is crucial in considering, starting, and maintaining therapy as it is defined “as the perceived capability to derive pathways to desired goals, and motivate oneself via agency thinking to use those pathways” (Snyder, 2002, p. 249). Therefore, treatment motivation depends on the individual level of suffering from symptoms and their consequences and the hope to be able to change something about them.

Sensing the necessity to change something and acknowledging the *need for psychological help* also depend on how one attributes the symptoms and their consequences (Schulte, 2015). However, a patient’s view on which aspects of their problems are internally or externally caused as well as perceptions of their stability, controllability, and situational variability, may obstruct one’s urge and perceived opportunities to change (Schulte, 2015). Having difficulties identifying, differentiating, and articulating feelings and emotions (alexithymia) might also impede internal attributions and has been shown to be associated with unfavorable treatment expectations (Schlechter et al., 2021; Terock et al., 2017). Furthermore, cultural norms may make it harder to seek psychological help, e.g., when psychological problems are stigmatized and perceived as weaknesses or cause embarrassment (Schlechter et al., 2021).

As treatment is supposed to lead to change, some patients may be concerned about potential negative effects of the treatment or about losing benefits resulting from symptom reduction, such as the loss of secondary illness gain (Schulte, 2015). Secondary illness gain can be external, like *symptom-related attention* from friends and family or a pension, or internal, like ego-defensive attributions that serve to maintain self-esteem and self-worth (Schulte, 2015). Therefore, the loss of secondary illness gain can make patients hesitant to consider treatment.

Furthermore, patients’ expectations about psychological treatment are greatly influenced by previous experiences and their general *knowledge about psychological treatment*. Patients may be more hesitant to start psychological treatment when they are unsure when to consider it, how to find help, how to pay for it, or what to expect in general (American Psychological Association APA, 2022). In contrast, if patients understand what psychological treatment is about and are aware of different treatment forms, it might facilitate considering and starting treatment.

Similarly, *initiative* and treatment motivation are closely related. In the work context, personal initiative is defined as a self-starting and proactive behavior that overcomes barriers to achieve a goal (Fay & Fay, 2001). Thus, initiative regarding treatment refers to taking action for one’s treatment, including actively seeking treatment and being generally optimistic regarding its success (Schulz et al., 1995, 2003). Individuals with high levels of initiative (i.e., intrinsic motivation) are more motivated to seek treatment and improve their mental health by actively seeking solutions and treatment options (Fishbach & Woolley, 2022; Westermann et al., 2019). Patients with higher levels of initiative are also more likely to adhere to treatment. In contrast, individuals with low initiative may need more encouragement and guidance from mental health professionals to overcome barriers and take steps toward recovery, i.e., extrinsic motivation (Fishbach & Woolley, 2022; Westermann et al., 2019).

Stewart et al. (2017) investigated whether motivation for psychological treatment predicted favorable outcomes in interdisciplinary multimodal pain treatment among patients with chronic pain. In this study, higher motivation for psychological treatment at intake predicted greater treatment benefits in pain intensity, depression, and global psychological distress (Stewart et al., 2017). In line with these findings, general treatment and outcome expectations have been shown to influence actual treatment success (Broderick et al., 2016; Burns et al., 2015; Cormier et al., 2016; Goossens et al., 2005). Above and beyond the general association between motivation and outcome, little is known about which components of psychological treatment motivation actually predict reductions in symptoms and distress. Therefore, this study aims to expand the findings of Stewart et al. (2017) by shedding more light on the different components of psychological treatment motivation that may predict favorable treatment outcomes. More detailed knowledge might help clinicians foster specific components of treatment motivation to more successfully treat their patients with chronic primary pain.

Stewart et al. (2017) used the total score of the German Questionnaire for Measuring Psychotherapy Motivation (FMP; Schneider et al., 1989). The FMP consists of four dimensions. However, factor analysis could not confirm these dimensions and suggests using only the total score (Schulz et al., 2003). Thus, to investigate the components of treatment motivation in more detail, the current study used the factor-analytically supported scales of the German Questionnaire for Psychotherapy Motivation (FPTM-23; Schulz et al., 2003) to achieve a more differentiated assessment of the components of treatment motivation. The scales of the FPTM-23 measure the components *hope*, *initiative*, *denial of the need for psychological help*, *knowledge of psychological treatment*, *symptom-related attention*, and *suffering* (for more details, see the method section).

Therefore, we expect that components potentially impeding psychological treatment like *denial of the need for psychological help* and *symptom-related attention* (i.e., secondary gain), predict unfavorable treatment outcomes. Moreover, we hypothesize that components that likely facilitate psychological treatment, such as *hope*, *initiative, knowledge of psychological treatment,* and *suffering,* predict lower levels of pain intensity, pain interference, depression, and anxiety after an interdisciplinary multimodal pain treatment.

## Methods

### Sample

The data of 294 patients with chronic primary pain were collected between December 2015 and May 2022. All patients fulfilled the diagnostic criteria of chronic primary pain (MG30.0) according to the ICD-11 (World Health Organization, 2019) and received inpatient care in a tertiary psychosomatic university clinic. Individuals were excluded if they were younger than 18 years, with insufficient German-language proficiency, and/or refused general consent to further use their data.

### Ethics Statement

This research has been approved by the ethics committee of the Canton of Bern, Switzerland (project ID 2018-00493, ID 2021-02214) and is in accordance with the Declaration of Helsinki. All patients were informed about the use of their data for research purposes, provided informed general consent, and signed informed consent regarding publishing anonymized data.

### Procedures

During the interdisciplinary multimodal pain treatment, patients completed psychometric assessments at intake and discharge for quality management purposes. During three 45-min psychometry sessions, patients completed a battery of self-reported questionnaires with the help of a research assistant. The FPTM-23 was part of this battery, which included questionnaires on the patient’s overall condition, psychopathological symptoms, clinically relevant behavior, and experience, as well as other treatment-related psychological constructs. On average, patients stayed for 23.3 days (*SD* = 4.9 days). The interdisciplinary multimodal treatment contained various treatment components (e.g., psychotherapy, medical interventions, pharmacotherapy, physiotherapy, and occupational therapy), which all patients were eligible to receive (Arnold et al., 2014). The psychological treatment followed a cognitive-behavioral treatment approach. The selection of treatment components within a treatment plan was individualized for each patient.

### Measures

Primary outcome measures for this research were defined following the “initiative on methods, measurement, and pain assessment in clinical trials” (IMMPACT) recommendations (Dworkin et al., 2005; Turk et al., 2008), as well as the “validation and application of a patient relevant core outcome set to assess effectiveness of multimodal pain therapy” (VAPAIN) consensus statement (Kaiser et al., 2018), i.e., changes in pain intensity, pain interference, and psychological functioning.

#### BPI

Pain intensity and pain interference were measured with the German version of the Brief Pain Inventory (BPI; Radbruch et al., 1999). The measure of pain intensity consists of four items with Likert scales ranging from no pain at all (0) to the worst pain imaginable (10). These four items assess the worst, least, average, and current pain and can be averaged to compute the pain intensity scale. Pain interference can be computed by averaging seven items, ranging from no interference (0) to complete interference (10). The resulting score for both scales ranges between 0 and 10. Radbruch et al. (1999) confirmed the two-factor structure of the BPI and showed good psychometric properties of the translated version. In the current sample, Cronbach’s alpha can be considered good for both pain intensity (0.90) and pain-related interference (0.81).

#### HADS-D

Psychological functioning, respectively, psychological distress during the past week was assessed using the German version of the Hospital Anxiety and Depression Scale (HADS-D; Petermann, 2011). The subscales anxiety and depression were measured with seven items each and are rated on four-point Likert scales from 0 to 3, leading to a possible score of 0–21 for each subscale. By adding the two subscales, a total score for psychological distress can be calculated. The German version of the HADS-D has good psychometric properties and the two-factor structure has been confirmed repeatedly (Herrmann-Lingen et al., 2011; Petermann, 2011). Cronbach’s alpha for this sample can be considered good, with 0.73 for the subscale of anxiety and 0.82 for the subscale of depression.

#### FPTM-23

The German short version of the questionnaire for psychotherapy motivation (FPTM-23, Schulz et al., 2003) measures the six scales *hope*, *initiative*, *denial of the need for psychological help*, *knowledge of psychological treatment*, *symptom-related attention*, and *suffering.* This questionnaire is considered to be particularly suitable for inpatients treated in psychosomatic medicine (Schulz et al., 2003). *Hope* includes statements about patients’ potential skepticism that the treatment will lead to relief and improvement (e.g., “I have the feeling that you cannot be helped here”). According to the test manual, all items of this scale are reverse-coded and had to be inverted prior to analysis. Thus, this scale rather assesses hopelessness and a lack of confidence. *Initiative* measures the patients’ efforts to obtain treatment, ranging from passivity to informing oneself and taking action (e.g., “The fact that I am here is due to my own endeavors.”). *Denial of the need for psychological help* encompasses loss of control, lack of character or independence, weakness of will as possible self-attributed reasons for needing psychological help, as well as the fear of losing face as a potential consequence of needing help (e.g., “people who have character can cope with their difficulties alone, without any help”). Knowledge and experience about or with psychological treatment before treatment are assessed with the scale *knowledge of psychological treatment* (e.g., “I have been in psychological/ psychotherapeutic treatment before”). The scale *symptom-related attention* assesses secondary illness gains that patients experience in their environment due to their condition, like increased understanding, assistance, or attention (e.g., “When I have my complaints, it happens more often than not that someone takes care of me.”). *Suffering* measures psychological distress and worries, as well as the desire and readiness to tackle one’s problems with the help of other people (e.g., “I can’t cope with my problems on my own any longer”). The questionnaire consists of 23 items. Each scale represents a sum score of 4 items, except for the scale for *symptom-related attention,* which is composed of 3 items. All items have a four Likert scale ranging from 1 “not true “ to 4 “true”. Internal consistencies for the individual scales in this inpatient sample can be considered satisfactory to good, as Cronbach’s alpha was 0.75 for *hope*, 0.82 for *initiative*, 0.72 for *denial of the need for psychological help*, 0.70 for *knowledge of psychological treatment*, 0.64 for *symptom-related attention*, and 0.80 for *suffering.* Apart from the scale for *symptom-related attention* ($\alpha$ = 0.78, Schulz et al., 2003), Cronbach’s alpha values were very similar to the reported values of Schulz et al. (2003). The FPTM-23 questionnaire was factor-analytically tested and the scales showed satisfactory psychometric characteristics in a similar sample of inpatients receiving psychological treatment in a psychosomatic ward (Schulz et al., 2003). However, there is limited evidence supporting the convergent and discriminant validity of the FPTM-23 questionnaire.

### Statistical Analyses

R and IBM SPSS Statistics (version 27) were used for statistical analyses (IBM Corp, 2020; R Core Team, 2021). Descriptive analyses were first computed for the demographic and clinical data. Paired *t*-tests were conducted to assess the changes between intake and discharge. The associations of the variables under investigation were measured by computing Pearson correlations. The significance level was set at *α* = 0.05 (two-tailed). The predictive value of the scales of psychological treatment motivation on anxiety, depression, pain intensity, and pain interference at post-treatment was investigated separately using hierarchical regression analyses. In a first step, the control variables age, gender, illness duration, as well as the initial level of each outcome variable were included in each regression analysis. In a second step, the scales of the FPTM-23 were added. Bonferroni correction was applied to correct for multiple comparisons of the six scales of the FPTM-23 as predictors of each treatment outcome. Multicollinearity was evaluated prior to analyzing the data using correlational analyses and the variance influence factor (VIF) (Alin, 2010; Daoud, 2017).

## Results

### Demographics and Clinical Data

The sample consisted of 294 patients with chronic primary pain. On average, patients were 44.8 years old (*SD* = 14.2 years; range = 18–81) and 61.9% were female. 47.3% of the patients reported suffering from pain between 1–5 years, and 28.2% for more than ten years. On average, patients reported pain intensity and interference between 5 and 6 on a scale of 0–10 at intake. Moreover, on average, patients reported elevated anxiety and depression levels (cut-off: > 8 for each subscale; Bjelland et al., 2002) at intake (see Table 2).

### Pre-treatment Analysis

Table 1 shows Pearson correlations of the FPTM-23 scales and outcome variables pre-treatment. Findings suggest that higher scores on the scale *hope* were significantly associated with lower values in pain intensity, pain interference, anxiety, and depression. *Suffering* was significantly and positively correlated with pain intensity, pain interference, anxiety, and depression. Values on the scale *denial of the need for psychological help* and *knowledge of psychological treatment* were positively associated with anxiety and depression. The scales *initiative* and *symptom-related attention* were not significantly correlated with any outcome variable.

**Table 1:** Correlations of study variables pre-treatment

		*M*	*SD*	1	2	3	4	5	6	7	8	9
1	FPTM-23 Hope	3.0	0.7									
2	FPTM-23 Initiative	2.6	0.9	.180**								
3	FPTM-23 Denial of the need for psychological help	1.7	0.7	− .163**	− 0.073							
4	FPTM-23 Knowledge of psychological treatment	2.8	0.9	− 0.032	.136*	− 0.020						
5	FPTM-23 Symptom-related attention	2.4	0.8	0.031	0.100	.159**	− 0.012					
6	FPTM-23 Suffering	2.7	0.8	− .339***	0.044	0.072	.262***	− 0.025				
7	BPI Pain intensity	5.3	1.8	− .174**	0.081	− 0.015	0.048	− 0.011	.143*			
8	BPI Pain interference	5.6	1.9	− .264***	0.006	0.074	0.071	0.012	.490***	.514***		
9	HADS-D Anxiety	10.0	4.1	− .326***	− 0.054	.188**	.130*	0.053	.582***	.189**	.418***	
10	HADS-D Depression	9.2	4.5	− .347***	− 0.012	.137*	.116*	− 0.029	.625***	.264***	.598***	.564***

*p < .05, **p < .01, ***p < .001

*M* mean; *SD* standard deviation; *FPTM-23* Questionnaire for Psychotherapy Motivation; *BPI* Brief Pain Inventory—German version; *HADS-D* Hospital Anxiety and Depression Scale—German version

### Comparisons Between Pre‑ and Post-Treatment

Table 2 summarizes mean values, standard deviation, paired *t*-tests, and effect sizes for different outcome measures at intake and discharge. Paired *t*-tests revealed that all outcome measures improved significantly over three weeks. Furthermore, 17% of patients in the current sample reported a reduction in pain intensity larger than 30%, which can be regarded as an at least moderate clinically relevant decrease during treatment according to the IMMPACT criteria. Moreover, 62% of all patients reported a reduction in pain interference by at least one unit on the NRS scale, indicating a clinically significant reduction (Dworkin et al., 2005). According to the reliable change index (RCI), 22% of all patients reported reliable changes from pre- to post-treatment in the subscale of anxiety. Similarly, 19% of patients reported reliable changes from pre- to post-treatment in the subscale of depression according to the RCI.

**Table 2:** Number of patients, mean, standard deviation, pre-post comparison, and effect size of different outcome measures

	N	Pre-treatment		Post-treatment		*t*	*d*
		*M*	*SD*	*M*	*SD*		
BPI Pain intensity	294	5.3	1.8	5.0	2.0	3.6***	0.205
BPI Pain interference	294	5.6	1.9	4.2	2.1	12.6***	0.734
HADS-D Anxiety	294	10.0	4.1	7.3	4.1	12.8***	0.749
HADS-D Depression	294	9.2	4.5	6.5	4.3	13.3***	0.777

*p < .05, **p < .01, ***p < .001

*N* number of patients; *M* mean; *SD* standard deviation; *t t* value; *d* Cohen’s *d*; *BPI* Brief Pain Inventory—German version; *HADS-D* Hospital Anxiety and Depression Scale—German version

### Regression Analyses

Tables 3 and 4 summarize the findings of the hierarchical linear regression analyses predicting mean pain intensity, pain interference, anxiety, and depression outcomes at the post-treatment assessment. Multicollinearity was unproblematic in all analyses (Alin, 2010; Daoud, 2017). The first step including age, sex, pain duration and pre-treatment outcome variable levels as control variables was significant for the prediction of pain intensity (F(4, 289) = 73.96, *p* < 0.001), pain interference (F(4, 289) = 38.57, *p* < 0.001), anxiety (F(4, 289) = 48.65, *p* < 0.001), and depression (F(4, 289) = 71.11, *p* < 0.001) outcomes post-treatment. In the second step, the scales of the FPTM-23 questionnaire were added. The second analysis reached significance for the prediction of pain intensity (F(10, 283) = 30.26, *p* < 0.001), pain interference (F(10, 283) = 18.49, *p* < 0.001), anxiety (F(10, 283) = 26.07, *p* < 0.001), and depression (F(10, 283) = 31.18, *p* < 0.001) outcomes post-treatment, and uniquely accounted for 1% for the variance in mean pain intensity, 5% in pain interference, 7% in anxiety, and 3% in depression after treatment. Findings suggest that higher values on the scale *hope* predicted lower levels of pain interference, anxiety, and depression post-treatment. Thus, lower levels of hopelessness seemed to predict lower levels of pain interference, anxiety, and depression post-treatment. Higher levels of *suffering* pre-treatment seemed to predict smaller decreases in anxiety post-treatment. None of the other FPTM-23 scales predicted post-treatment levels of pain intensity, pain interference, anxiety, and depression post-treatment. Considering the six scales from the FPTM-23 questionnaire in the regression analysis, Bonferroni correction was applied and the adjusted *p* < 0.008 marked statistical significance. Even after correcting for Bonferroni, the changes in the scales *hope* and *suffering* remained significant (*p* < 0.001).

**Table 3:** Hierarchical regression analysis predicting mean pain intensity and pain interference (BPI) outcomes at the post-treatment assessment

	BPI Pain intensity (step 2: R^2^ = 0.51; adj. R^2^ = 0.50)					BPI Pain interference (step 2: R^2^ = 0.40; adj. R^2^ = 0.37)				
	*B*	*SE*	$\beta$	*t*	*R*^*2*^*ch*	*B*	*SE*	$\beta$	*t*	*R*^*2*^*ch*
Step 1: Control variables					0.50***					0.35***
Step 2: FPTM-23 scales					0.01					0.05**
Control variables										
Age	− 0.01	0.01	− 0.04	− 0.85		− 0.01	0.01	− 0.07	− 1.45	
Sex	0.09	0.18	0.02	0.50		− 0.16	0.21	− 0.04	− 0.75	
Pain duration	0.14	0.07	0.09	1.96		0.15	0.08	0.09	1.76	
Pre-treatment outcome levels	0.76	0.05	0.70	16.05***		0.55	0.06	0.51	9.46***	
FPTM-23 scales										
Hope	− 0.24	0.13	− 0.09	− 1.86		− 0.63	0.15	− 0.21	− 4.14***	
Initiative	0.01	0.09	0.01	0.03		− 0.02	0.11	− 0.01	− 0.16	
Denial of the need for psychological help	− 0.18	0.13	− 0.06	− 1.36		− 0.02	0.15	− 0.01	− 0.12	
Knowledge of psychological treatment	− 0.06	0.10	− 0.03	− 0.59		− 0.04	0.12	− 0.02	− 0.37	
Symptom-related attention	− 0.07	0.10	− 0.03	− 0.73		− 0.13	0.12	− 0.05	− 1.05	
Suffering	− 0.04	0.11	− 0.02	− 0.35		0.03	0.15	0.01	0.20	

*p < .05, **p < .01, ***p < .001

*B* unstandardized beta; *SE* standard error; $\beta$ standardized beta; *t t* value; *R*^*2*^*ch R*^*2*^ change

**Table 4:** Hierarchical regression analysis predicting mean anxiety and depression (HADS-D) outcomes at the post-treatment assessment

	HADS-D Anxiety (step 2: R^2^ = 0.48; adj. R^2^ = 0.46)					HADS-D Depression (step 2: R^2^ = 0.52; adj. R^2^ = 0.51)				
	*B*	*SE*	$\beta$	*t*	*R*^*2*^*ch*	*B*	*SE*	$\beta$	***t***	***R***^***2***^***ch***
Step 1: Control variables					0.40***					0.50***
Step 2: FPTM-23 scales					0.07***					0.03*
Control variables										
Age	− 0.01	0.01	− 0.05	− 1.06		− 0.01	0.01	− 0.04	− 0.99	
Sex	0.15	0.38	0.02	0.39		− 0.39	0.39	− 0.04	− 1.01	
Pain duration	0.25	0.15	0.07	1.62		0.27	0.16	0.07	1.73	
Pre-treatment outcome levels	0.44	0.05	0.44	8.11***		0.56	0.05	0.58	10.75***	
FPTM− 23 scales										
Hope	− 1.22	0.28	− 0.21	− 4.35***		− 0.84	0.29	− 0.14	− 2.95**	
Initiative	0.09	0.20	0.02	0.45		− 0.08	0.20	− 0.02	− 0.38	
Denial of the need for psychological help	0.12	0.27	0.02	0.44		0.04	0.27	0.01	0.16	
Knowledge of psychological treatment	− 0.22	0.22	− 0.05	− 0.99		− 0.02	0.22	0.01	− 0.09	
Symptom-related attention	0.17	0.22	0.03	0.78		− 0.08	0.22	− 0.02	− 0.36	
Suffering	1.02	0.28	0.20	3.59***		0.58	0.30	0.11	1.96	

*p < .05, **p < .01, ***p < .001

*B* unstandardized beta; *SE* standard error; $\beta$ standardized beta; *t t* value; *R*^*2*^*ch R*^*2*^ change

## Discussion

This study aimed to evaluate outcome prediction in an interdisciplinary multimodal inpatient treatment of patients with chronic primary pain by different components of psychological treatment motivation. We hypothesized that components potentially impeding psychological treatment, like *denial of the need for psychological help* and *symptom-related attention,* predict unfavorable treatment outcomes. Furthermore, we hypothesized that motivational components that potentially facilitate psychological treatment, like *hope*, *initiative, knowledge of psychological treatment,* and *suffering,* predict lower levels of pain intensity, pain interference, depression, and anxiety after treatment.

We found that (1) pain intensity, pain interference, anxiety, and depression levels were significantly reduced after treatment. However, (2) none of the scales of treatment motivation predicted pain intensity levels after treatment. Furthermore, (3) neither *denial of the need for psychological help* nor *symptom-related attention* predicted unfavorable treatment outcomes, nor did (4) *initiative* or *knowledge of psychological treatment* predict post-treatment levels of pain interference, anxiety, and depression after treatment. Also, against our hypothesis, (5) higher levels of *suffering* at intake predicted smaller decreases in anxiety after treatment. In line with our hypothesis, we found that (6) lower levels of hopelessness as a component of treatment motivation predicted lower levels of pain interference, anxiety, and depression after treatment.

Since all outcome measures improved after treatment, our findings generally support the suitability of an interdisciplinary multimodal inpatient treatment of chronic primary pain consisting of various interventions to increase physical and psychological functioning (Kaiser et al., 2018). Whereas Stewart et al. (2017) have generally shown that psychological treatment motivation predicted favorable outcomes in an interdisciplinary multimodal pain treatment, it is less clear which components of treatment motivation are particularly predictive and should be fostered for successfully treating patients with chronic pain.

First of all, we did not find any component of psychological treatment motivation that predicted pain intensity levels post-treatment. Even though patients’ pain intensity reduced significantly over three weeks, the effect was small. This is not surprising since the majority of this sample’s patients had suffered from pain and the associated impairments for several years. Since large reductions in pain intensity among chronic pain patients after treatment are rather improbable (Kaiser et al., 2018), interdisciplinary multimodal pain treatment usually focuses more on increasing physical and psychological functioning and therefore reducing pain interference and psychological distress as opposed to reducing pain intensity (Kaiser et al., 2018).

Contrary to our overarching hypothesis that motivational components might facilitate or impede psychological treatment, we could not show that the motivational components *denial of the need for psychological help* or *symptom-related attention* predicted unfavorable treatment outcomes. As patients’ scores for *denial of the need for psychological help* in this sample were relatively low, one might argue that patients who cannot acknowledge the need for psychological help do not even consider or participate in interdisciplinary multimodal pain treatment. The same could be true for symptom-related attention. Since the sample consists only of patients who considered and started treatment, the fear of losing secondary illness gain does not seem to be a primary concern among these patients. In fact, it could even be that patients in this sample might even hope for secondary illness gains from the inpatient setting and care. These factors might explain why *denial of the need for psychological help* and *symptom-related attention* were not predictive of unfavorable treatment outcomes, as they might be more important for considering treatment.

Moreover, we were unable to show that the motivational components *initiative, knowledge of psychological treatment,* and *suffering* predicted lower post-treatment levels of pain intensity, pain interference, depression, and anxiety. Even though *initiative* measures patients’ efforts to obtain treatment and ranges from passivity to informing oneself and taking action, it could be that patients’ assessment of their *initiative* depends more on the healthcare system than on themselves. Consequently, patients’ self-reported *initiative* might be reduced, as patients need to be referred by their primary care physician or specialist for interdisciplinary multimodal pain treatment. Moreover, previous findings show that especially internal beliefs of health control seem predictive of treatment outcomes among patients with chronic pain, whereas external beliefs of health control were not (Zuercher-Huerlimann et al., 2019). Thus, because personal beliefs seem particularly important for pain patients, the *initiative* scale may not be ideal for assessing relevant aspects of chronic pain patients’ initiative.

As patients’ expectations about psychological treatment are greatly influenced by previous experiences and their general knowledge about psychological treatment, we were surprised that *knowledge of psychological treatment* did not predict favorable treatment outcomes. A closer look at the individual items revealed that most patients had already received psychological treatment and thus had experience with psychotherapy. It should be noted that previous experiences have not necessarily been positive and may depend on the perceived outcome of this prior treatment. Moreover, very few had acquired theoretical knowledge about psychological treatment. Because these different trends were averaged for scaling, it may be that this results in a loss of predictive value for treatment success. In general, one might argue that *initiative* and *knowledge of psychological treatment* may influence considering and seeking an inpatient treatment in the first place but might be less relevant later within the inpatient treatment.

Interestingly, *suffering* at intake predicted smaller decreases in anxiety after treatment. Suffering is often viewed as a driving force of treatment as it measures psychological distress and worries, as well as the desire and readiness to tackle one’s problems with the help of other people (Schulte, 2015; Schulz et al., 2003). Similarly, patients with high suffering values could not benefit more from the treatment than patients with lower suffering values during treatment. Thus, *suffering* seemed to mainly serve as motivation to seek treatment instead of predicting treatment effects among patients with chronic primary pain.

In line with our hypothesis, the scale of *hope* predicted favorable treatment outcomes. Thus, as the items of this scale rather assess hopelessness, these findings suggest that lower levels of hopelessness and, therefore, less skepticism toward the treatment predicted favorable treatment outcomes in pain interference, anxiety, and depression. As not being hopeless does not necessarily mean being full of hope, it cannot be concluded from our results that the patients’ level of hope predicted treatment outcomes. This kind of reasoning aligns with previous research, showing that hopelessness and hope are distinct but correlated constructs (Huen et al., 2015). Whereas cognitive-behavioral interventions have been shown to reduce hopelessness among older adults (Hernandez & Overholser, 2021), in our patients, hope might also be fostered by psychoeducation and providing general knowledge about the treatment. Along these lines, Larsen et al. (2015) were able to show that participants with chronic pain experienced and sustained hope through a group treatment intervention called “hope and strengths activity”. By focusing on their strengths, participants were able to foster personal hope by comparing themselves to others, taking perspective, experiencing communion, and connecting with other group members (Larsen et al., 2015). Also, Cheavens et al. (2006) found that hope-based group therapy helped participants from a community sample learn how to set meaningful and realistic goals, develop strategies to achieve those goals, identify sources and drains of motivation, and adapt their goals and strategies if needed. Therefore, hope-based interventions may be particularly suited to be implemented in interdisciplinary multimodal pain treatment to improve treatment outcomes.

Overall, our findings suggest that above and beyond providing symptom relief, reducing hopelessness, as well as fostering hope regarding treatment process and outcome might help clinicians to treat patients with chronic primary pain more effectively.

## Limitations and Future Research

Several limitations need to be addressed. This study is not representative of all our inpatients with chronic pain since consent was required to analyze patients’ data. In addition, our sample was recruited in a single center in one country, included only patients with primary chronic pain, and may not be representative of all inpatients with chronic pain. Even though interdisciplinary multimodal pain treatment has been shown to be particularly suitable for treating chronic pain, the current study design does not allow for any conclusions about the sustainability of effects nor the direction of causality, as data was only assessed before and after treatment. Furthermore, it was not possible to control for or manipulate all potentially confounding variables because interdisciplinary multimodal pain treatment includes different forms of therapy and is tailored individually for each patient. Thus, it remains unclear if and how treatment motivation might have influenced patients’ engagement in each treatment modality within the interdisciplinary multimodal pain treatment and may potentially link to outcomes.

Future studies might benefit from longitudinal designs to draw conclusions on causality and make predictive statements on the sustainability of the effects after treatment. Moreover, larger samples will be necessary to disentangle potential mediating effects of motivational components facilitating or hindering treatment. Furthermore, studying the relationship between psychological treatment motivation and treatment outcome in an ambulatory setting might be interesting, as some components of psychological treatment motivation seem more relevant in considering and seeking treatment. As hopelessness and hope are two distinct but correlated constructs (Huen et al., 2015), future studies are needed to replicate these findings with other questionnaires since the labeling of the FPTM-23 scales might be misleading regarding *hope*, as the items rather assess hopelessness*.*

## Conclusions

Taken together, *hope(lessness)* and *suffering* as components of psychological treatment motivation seemed to predict changes in pain interference, anxiety, and depression after interdisciplinary multimodal pain treatment, as *hope*, i.e., lower levels of hopelessness, predicted lower values in pain interference, anxiety, and depression post-treatment. *Suffering* seemed to predict smaller decreases in anxiety post-treatment. Especially fostering *hope* regarding the treatment might help clinicians successfully treat patients with chronic primary pain.

## Data Availability

The data stems from inpatients with chronic primary pain who have received interdisciplinary multimodal pain treatment at the Inselspital Bern.
